# mTOR inhibition suppresses Myc-driven polyposis by inducing immunogenic cell death

**DOI:** 10.1038/s41388-023-02706-6

**Published:** 2023-05-03

**Authors:** Brian J. Leibowitz, Guangyi Zhao, Wenxin Xia, Yuhan Wang, Hang Ruan, Lin Zhang, Jian Yu

**Affiliations:** 1 Department of Pathology, University of Pittsburgh School of Medicine, UPMC Hillman Cancer Center, Pittsburgh, PA 15213 USA; 2 Department of Radiation Oncology, University of Pittsburgh School of Medicine, UPMC Hillman Cancer Center, Pittsburgh, PA 15213 USA; 3 Department of Pharmacology and Chemical Biology, University of Pittsburgh School of Medicine, UPMC Hillman Cancer Center, Pittsburgh, PA 15213 USA; 4 Present Address: Department of Biochemistry and Molecular Pharmacology at New York University Grossman School of Medicine, New York, NY 10016 USA; 5 Present Address: Department of Medicine, University of Southern California, Keck School of Medicine, Los Angeles, CA 90033 USA

**Keywords:** Cancer, Genetics

## Abstract

Myc is a key driver of colorectal cancer initiation and progression, but remains a difficult drug target. In this study, we show that mTOR inhibition potently suppresses intestinal polyp formation, regresses established polyps, and prolongs lifespan of *APC*^*Min/+*^ mice. Everolimus in diet strongly reduces p-4EBP1, p-S6, and Myc levels, and induces apoptosis of cells with activated β-catenin (p-S552) in the polyps on day 3. The cell death is accompanied by ER stress, activation of the extrinsic apoptotic pathway, innate immune cell recruitment, and followed by T-cell infiltration on day 14 persisting for months thereafter. These effects are absent in normal intestinal crypts with physiologic levels of Myc and a high rate of proliferation. Using normal human colonic epithelial cells, *EIF4E S209A* knockin and *BID* knockout mice, we found that local inflammation and antitumor efficacy of Everolimus requires Myc-dependent induction of ER stress and apoptosis. These findings demonstrate mTOR and deregulated Myc as a selective vulnerability of mutant *APC*-driven intestinal tumorigenesis, whose inhibition disrupts metabolic and immune adaptation and reactivates immune surveillance necessary for long-term tumor control.

## Introduction

Colorectal cancer (CRC) is the second leading cause of cancer deaths worldwide, with a five-year survival rate of 11% for metastatic disease [1]. Mutational loss of the tumor suppressor *APC* serves as the major initiating event in over 85% of sporadic CRC and leads to unrestrained activation of Wnt signaling and elevated expression of Myc. Constitutive Wnt signaling works in concert with elevated Myc and acquired mutations in MAPK and/or PI3K/mTOR pathways to promote CRC initiation and progression [2]. CRC treatment includes 5-fluorouracil (5-FU)-based chemotherapy and targeted therapies with specific genetic indications. The response rate to chemotherapy is roughly 10–15% in advanced disease [3]. EGFR antibodies or mutant Braf inhibitors are only used in wildtype (WT) *KRAS* CRC patients with a modest and transient response usually lasting several months [4]. Immune checkpoint blockade (ICB) therapies targeting T-cell activation offer promise for patients that did not respond to standard therapies; however the success is limited in most solid tumors such as CRC that are microsatellite stable [5, 6]. Wnt/Myc signaling promotes proliferation and alters tumor cell metabolism and the tumor microenvironment (TME) [7, 8]. These findings support that tumor cells play a significant role in shaping the immunosuppressive landscape [9–11]. CRC development often takes decades, and targeting critical drivers such as Myc can have a significant impact on tumor cells and their co-evolution with the TME. Despite intensive interest and efforts, currently there is no FDA-approved agent directly targeting Myc [12].

Cancer development is associated with chronically elevated stress due to DNA replication, protein synthesis, folding and quality control, nutrient and oxygen deficiency, as well as inflammation [13]. These stresses are sensed by distinct kinases and converge on the endoplasmic reticulum (ER) and phosphorylation of eIF2α (p-eIF2α) [14]. A mild increase in p-eIF2α and ER stress promotes adaptation and cell survival through suppression of global cap-dependent translation, and induction of transcription and translation of numerous genes involved in metabolism, protein folding and degradation, and antioxidant defense. Prolonged p-eIF2α elevation indicates failure in adaptation and leads to apoptosis through CHOP-dependent expression of death effectors such as DR5, PUMA and Noxa in the extrinsic and intrinsic pathways [14]. Myc-driven cancers are characterized by metabolic and translational stress coupled through GCN2-p-eIF2α-ATF4 signaling [15, 16]. Interestingly, Myc translation is regulated by eIF4E and 4EBP1 downstream of mTOR and ERK signaling [17]. Everolimus and Temsirolimus are allosteric inhibitors of mTOR and FDA-approved for therapy-refractory renal cancer. Their antitumor activity is primarily associated with suppression of tumor cell growth and angiogenesis through well-characterized targets such as 4EBP1 and S6K1 [18]. mTOR inhibitors can induce apoptosis in CRC cells and xenografts through ER stress and the extrinsic pathway [19, 20].

Immunogenic cell death (ICD) is a type of cell death that primes a systemic immune response, and plays a critical role in the host defense against viral and bacterial infection [21]. ICD is associated with several hallmarks, such as elevated p-eIF2α, cell-surface exposure of the ER chaperone calreticulin (CRT or CLAR), extracellular release of damage-associated molecular patterns (DAMPs), production of cytokines, and activation of the Type 1 interferon response associated with nucleic acid sensing. Subsequent recruitment and activation of neutrophils, macrophages, dendritic cells (DCs), and Natural Killer cells (NKs) is mediated by pattern recognition receptors (PRRs), antigen processing and presentation, and accompanied by timely production of chemokines and inflammatory cytokines. This finally leads to cross-priming and activation of CD8+ lymphocytes and killing of infected cells, and the development of T-cell-mediated memory. ICD is a complex process, involving many immune cell types and mediators in the innate and adaptive compartments [21]. It is also induced by various anticancer agents, while detailed mechanisms, major mediators, and in vivo outcomes appear context-dependent and not well understood [21, 22].

In this study, we explored if elevated Myc and proteostress can serve as a druggable vulnerability in CRC initiation and the potential effects in the TME. Utilizing Everolimus, the *APC*^*Min/+*^ mouse model crossed with *EIF4E S209A* knockin and *BID* knockout strains, and normal human colonic epithelial cells, we demonstrated that mTOR inhibition selectively induces ER stress and apoptosis in Myc-high cells caused by *APC* loss, which promotes a cell-death-dependent local immune response and tumor control. These data support a vital role of mTOR in Myc-driven metabolic and immune adaptation, and as a highly specific vulnerability in the early stage of CRC development.

## Results

### Everolimus prevents and regresses polyposis in *APC*^*Min/+*^ mice

*APC*^*Min/+*^ mice are a widely used model to study Myc-driven oncogenesis, and our recent work showed that Myc translation is controlled by p-4EBP1 (S65/70) and p-eIF4E (S209) downstream of mTOR and ERK signaling [19]. We first determined if Everolimus can prevent polyposis. *APC*^*Min/+*^ mice fed with high-fat AIN-93G diet starting at week 4 died by week 31 with numerous intestinal polyps [23, 24]. Adding Everolimus (37.5 mg/kg) in the diet significantly prolonged their lifespan, with all mice alive at week 48 (Fig. S1A, B). By week 12, *APC*^*Min/+*^ mice had 25–45 macroscopic adenomas in the small intestine and colon (>1 mm diameter), which were reduced by around 90% with Everolimus (Fig. S1C–E).

**Fig. 1:**
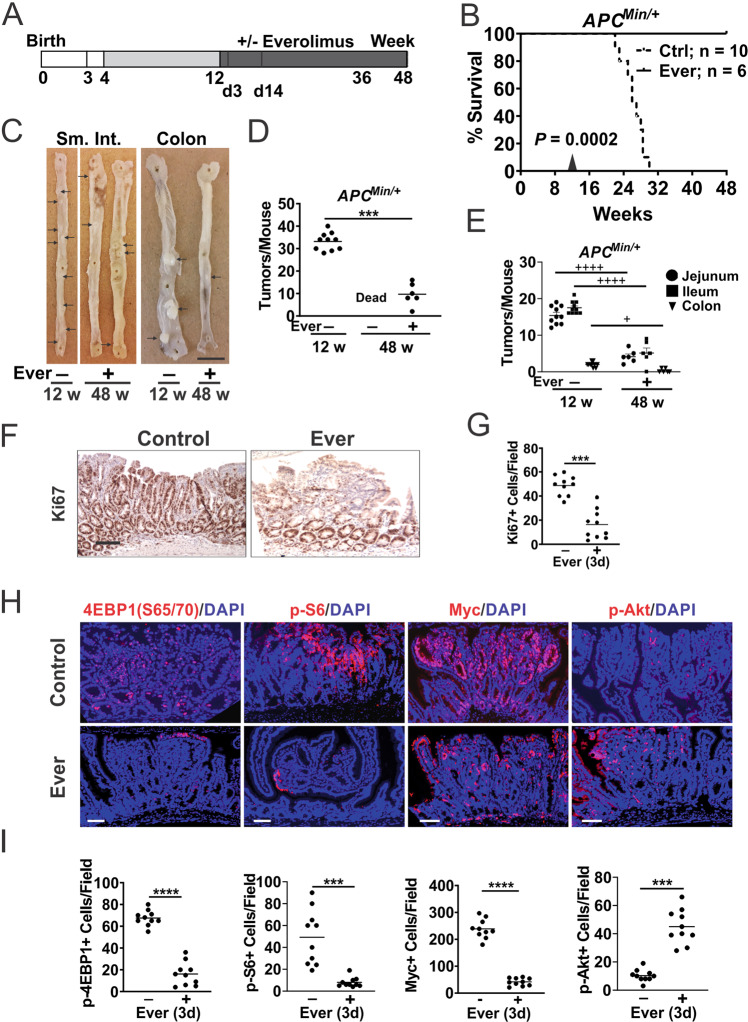
Everolimus regresses established intestinal polyps in *APC*^*Min*/+^ mice. *APC*^*Min/+*^ mice were fed control (Ctrl) diet from 4–12 weeks to established polyps followed by Ctrl diet with or without Everolimus (Ever) till week 48. **A** Schematic of polyp establishment, treatment, and harvest. The small intestine (SI), colon and polyps were analyzed on week 12 (12 w), day 3 or 14 of the treatment (3d, 14d later), and week 48 (48 w, 36 w treatment). **B** Kaplan–Meier curve of *APC*^*Min*/+^ mice at 48 w. Log-Rank test. **C** Representative images of whole mount small intestine and colon from mice. Bar = 1 cm. Polyps are indicated with arrows. **D** Quantitation of total macroscopic adenomas from (**C**). **E** Quantitation of macroscopic adenomas in different regions from (**C**). *n* = 10 or 6 mice/group. **F** Representative Ki67 IHC staining at 3d. Bar = 100 µm. **G** Quantitation of positive cells per 400× field from (**F**). **H** Representative IF staining of the indicated proteins in polyps at d3. Bars = 100 µm. **I** Quantitation of positive cells per 400× field from (**H**). Nuclear Myc was scored. **D**, **E** *n* = 6–10 mice/group. **G**, **I**, *n* = 3 mice/group, and 3–4 randomly chosen polyps/mouse. ***P < 0.001, ****P < 0.0001 (Student’s *T*-Test, two-tailed). ^+^*P* < 0.05, ^++++^*P* < 0.0001 (One-way ANOVA and Tukey Post-Hoc test).

Next, we assessed if Everolimus is effective against established polyps. *APC*^*Min/+*^ mice on control diet up to week 12 were randomly divided in two groups to receive diet with Everolimus (37.5 mg/kg) or without (Fig. 1A). All mice in the Everolimus group were alive at week 48 while all in the control group died by week 31 (Fig. 1B). The Everolimus group did not have obvious health issues and showed decreased tumor burden by approximately 75% from week 12 (Fig. 1C, D). The reduction was observed in the small intestine and colon (Fig. 1E).

To assess the acute response to Everolimus, we harvested the intestine with established polyps after 3 days of treatment. As expected, Everolimus strongly reduced cell proliferation (Ki67+) in the polyps, but not in the morphologically “normal” intestinal crypts (Fig. 1F, G). Consistent with on-target mTOR inhibition by Everolimus, p-4EBP1, p-S6, and nuclear Myc levels significantly decreased in the polyps (Fig. 1H, I). p-Akt (S473) strongly increased (by around fourfold) (Fig. 1H, I), which is a known feedback response due to loss of S6K1-mediated inhibition of IRS/PI3K [20, 25]. In contrast, “normal” intestinal crypts were highly proliferative with nuclear Myc (Ki67+; Fig. S2A), but very lower basal mTOR/AKT markers (up to 100-fold) compared to the polyps (Fig. S2B, C). Slightly increased levels of p-4EBP1 and p-AKT, but no significant changes in nuclear Myc or p-S6 were detected in the crypts on day 3 (Fig. S2B, C). Using a different antibody, we detected striking elevation of cytoplasmic Myc in the polyps compared to the crypts, which was reduced by Everolimus treatment similarly to nuclear Myc (Fig. S2F–H). These data demonstrate that mutant *APC*-driven polyps deregulate Myc and p-4EBP1 and are highly sensitive to mTOR inhibition with a strong feedback AKT activation.

### Everolimus induces ER stress and apoptosis in the polyps

**Fig. 2:**
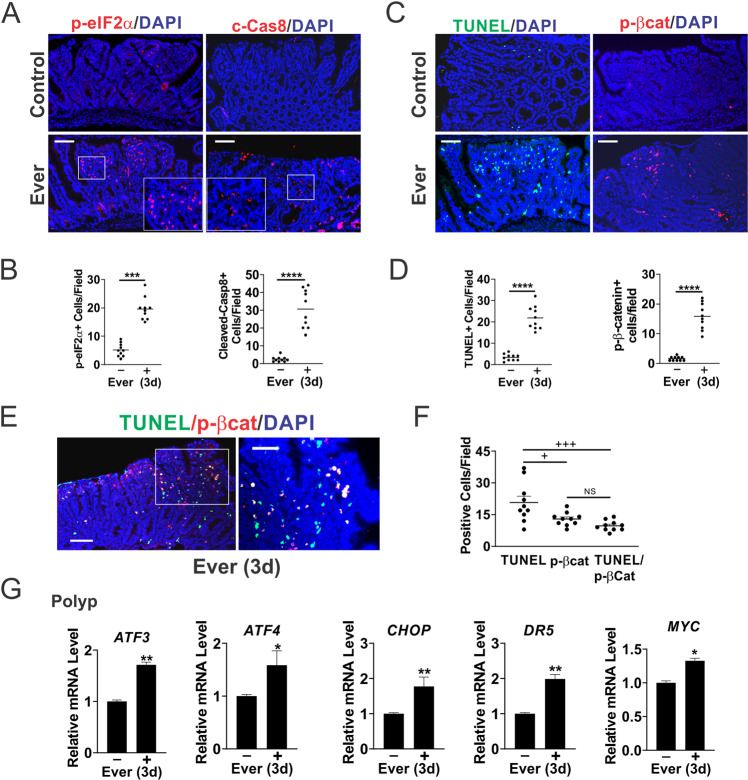
Everolimus selectively induces apoptosis and ER stress in the polyps. *APC*^*Min/+*^ mice with established polyps were treated with Everolimus for 3 days [3d]. Small intestinal polyps were analyzed. **A** Representative phospho-eIF2α (S51, p-eIF2α) and Cleaved-caspase-8 (c-Cas8) IF staining in the polyps. Higher magnification images are shown in insets. Bar = 100 µm. **B** Quantitation of p-eIF2α+ and c-Cas8+ per 400× field from A. Bar = 100 µm. **C** Representative TUNEL and phospho-β-catenin (S552) (p-βcat) IF staining. Bar = 100 µm. **D** Quantitation of TUNEL+ and p-βcat+ cells per 400× field from (**C**). **E** Representative TUNEL/p-βcat double IF staining. Bar = 100 µm. **F** Quantitation of cells per 400× field from (**E**). **G** qRT-PCR analysis of the indicated genes. cDNA was made from pooled polyps, 3–5 polyps/mouse, and 3 mice/group. **B**, **D**, **F**, **G**, *n* = 3 mice/group. *P < 0.05, **P < 0.01, ***P < 0.001, ****P < 0.001 (Student’s *T*-test, two-tailed). ^+^*P* < 0.05, ^+++^*P* < 0.0001 (one-way ANOVA and Tukey Post-Hoc test).

Regression of established polyps cannot be explained by either reduced proliferation or AKT activation. Our previous work reported that mTOR inhibitors and glutamine starvation induce ER stress-dependent apoptosis of CRC cells [20, 22]. We therefore examined ER stress and cell death in the polyps. p-eIF2α (s51) and cleaved-caspase-8 were highly elevated (up to tenfold) 3 days after Everolimus treatment (Fig. 2A, B). Increased cell death (near 20-fold) was also confirmed by TUNEL, along with highly (over tenfold) elevated C-terminal phosphorylation of β-catenin (S552, p-βcat), a target of AKT during intestinal stem cell activation [24, 26, 27] (Fig. 2C, D). Double staining revealed a strong overlap of TUNEL and p-βcat, with over 70% of p-βcat+ cells positive for TUNEL, and 50% of TUNEL+ cells positive for p-βcat (Fig. 2E, F). In contrast, all three markers showed little or no change upon treatment in normal intestinal crypts compared to polyps (Fig. S2D, E). A slight increase in TUNEL is likely due to occasional crypt cells with elevated p-AKT and spontaneous *APC* loss. qRT-PCR confirmed the induction of ER stress markers (*ATF3, ATF4, CHOP*, and *DR5*) in the polyps (Fig. 2G). *MYC* mRNA increased slightly (Fig. 2G), while Myc protein decreased (Fig. 1H, I). These data indicate that Everolimus treatment induces acute ER stress and cell death in the context of mutant *APC*, suggesting that elevated Myc leads to the vulnerability to mTOR inhibition.

### Everolimus promotes rapid immune activation in the TME

**Fig. 3:**
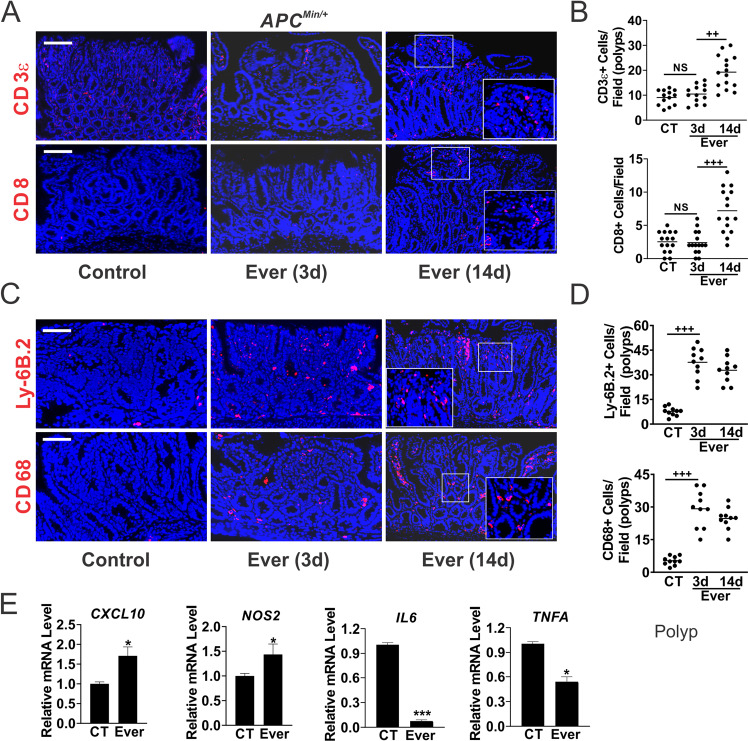
Everolimus induces innate immune cell infiltration in the polyps. *APC*^*Min/+*^ mice with established polyps were treated with Everolimus for 3 days or 14 days (Fig. 1A). The intestinal polyps were analyzed. **A** Representative CD3ε and CD8 IF staining in the polyps. Bar = 100 µm. **B** Quantitation of cells per 400× field from (**A**). **C** Representative Ly-6B.2 and CD68 IF staining. Higher magnification images are shown in insets (**A**, **C**). **D** Quantitation of cells per 400× field from (**D**). **E** qRT-PCR analysis of the indicated immune genes. cDNA was made from pooled polyps, 3–5 polyps/mouse, and 3 mice/group. **B**, **D**, **E**, *n* = 3 mice/group. *P < 0.05, ***P < 0.001 (Student’s *T*-Test, two-tailed). ^+++^*P* < 0.001 (one-way ANOVA and Tukey Post-Hoc test).

The TME co-evolves with the tumor gradually towards immunosuppression in order to avoid immune detection or destruction, or immune stress [22]. We found very few CD3-epsilon (CD3, thereafter) + cells, and virtually no CD8+ cells in the polyps from *APC*^*Min/+*^ mice at week 12. Their numbers did not change on day 3 of Everolimus treatment but increased significantly on day 14 (Fig. 3A, B), nor the expression of *CD4* or *CD8* assessed by RT-PCR (Fig. S3A). There were a few innate cells such as macrophages (CD68+) and neutrophils (Ly-6B.2+) in the polyps from the control group, but their numbers increased on day 3 of Everolimus treatment and stayed elevated on day 14 (up to 7-fold) (Fig. 3C, D). TME remodeling was already evident on day 3, including increased expression of proinflammatory macrophage M1 markers *CXCL10* and *NOS2*, decreased expression of M2 markers *Trem2* and *Arginase*, as well as T-cell inhibitory markers *IL6, TNFA, PD1*, and *PDL1* in the polyps (Figs. 3E and S3A). In the Everolimus group at week 48, only a few residual polyps were detected with highly elevated CD8+, neutrophils and macrophages, indicating long-term antitumor immunity (Fig. S3B). These data suggest that metabolic and immune adaptation in CRC development can be efficiently targeted by mTORi.

### *APC* loss confers Myc-dependent sensitivity to Everolimus

**Fig. 4:**
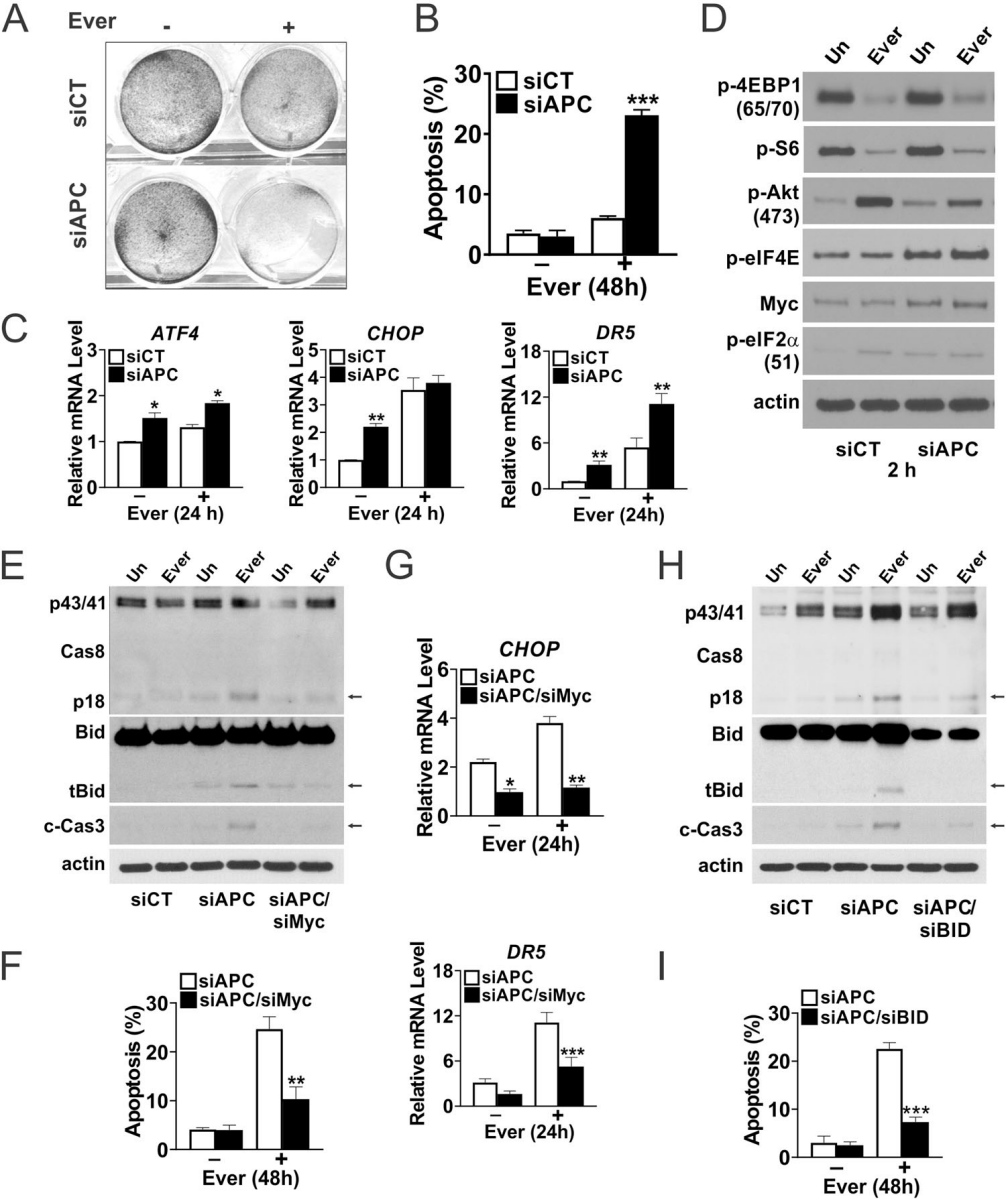
Everolimus activates the extrinsic pathway in human colonic epithelial cells upon *APC* loss. Human NCM356 cells were transfected with control or the indicated siRNA duplexes and treated with Everolimus (10 μm) for the indicated times. **A** Attached cells at 48 h were visualized by crystal violet staining. **B** Apoptosis was measured at 48 h by Hoechst staining of fragmented nuclei. **C** qRT-PCR analysis of the indicated genes at 24 h. **D** Western blots of the indicated proteins at 2 h. **E** Western blots of the indicated proteins at 24 h and **F** Apoptosis at 48 h measured by Hoechst staining of fragmented nuclei with Myc siRNA. **G** qRT-PCR analysis of the indicated genes at 24 h with Myc siRNA. **H** Western blots of the indicated proteins at 24 h and **I** apoptosis at 48 h measured by Hoechst staining of fragmented nuclei with *BID* siRNA. Arrows indicated cleaved bands. **B**, **C**, **F**, **G**, **I**, *n* = 3 independent experiments. *P < 0.05, **P < 0.01, ***P < 0.001 (Student’s *T*-Test, two-tailed).

To better understand the selectivity of mTORi, we transfected NCM356 normal human colonic cells with control or a previously validated *APC* siRNA [23]. *APC* siRNA increased the sensitivity of NCM356 cells to Everolimus-induced cell death by fivefold at 48 h (Figs. 4A, B, and S4A), with increased basal expression of ER stress genes *ATF4*, *CHOP*, and *DR5* and at 24 h (Fig. 4C). *APC* siRNA increased basal levels of several ER markers and TNFR family members as well (Fig. S4B). *APC* siRNA also increased basal Myc, p-AKT, p-eIF4E, and p-eIF2α, mimicking elevated mTOR and ER stress in the polyps. Consistent with findings in the polyps, Everolimus showed on-target p-4EBP1 and p-S6 reduction and p-AKT increase in NCM356 cells within 2 h (Fig. 4D).

We then tested if elevated Myc mediates the sensitivity to mTORi upon *APC* knockdown. *MYC* siRNA suppressed cleavage of caspase-8, Bid (tBid), and caspase-3, and induction of apoptosis (by 60%), *CHOP* and *DR5* (Figs. 4E–G and S4B). Truncated Bid (tBid) is generated by caspase-8, and amplifies death receptor signaling via mitochondrial damage and caspase-9/-3 activation [28, 29]. *BID* siRNA significantly blocked caspase-3 activation and apoptosis but not caspase-8 cleavage (Fig. 4H, I). siRNA knockdown efficiency and western blots were also quantified (Fig. S4D). These data indicate that *APC* loss creates a Myc-dependent vulnerability to mTOR inhibition via enhanced ER stress and activation of the extrinsic apoptotic pathway.

### Everolimus-induced cell death and immune recruitment in the polyps is dependent on Myc protein

**Fig. 5:**
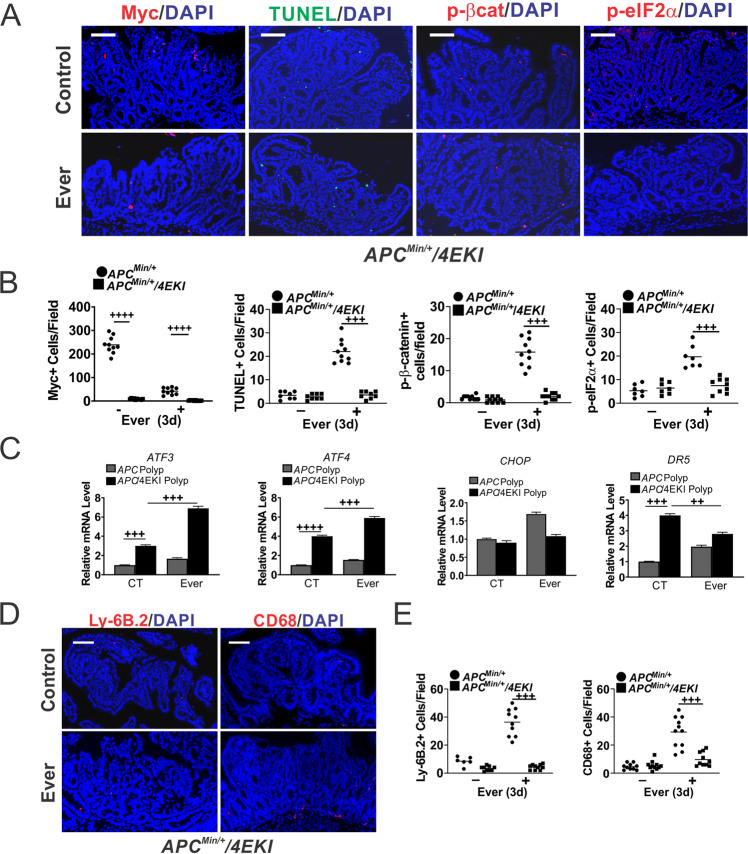
Everolimus-induced Myc-dependent cell death and immune infiltrations in the polyps. *APC*^*Min/+*^ or *APC*^*Min/+*^*/4ES209A (KI)* mice with established polyps were treated with Everolimus for 3 days. The intestinal polyps were analyzed. **A** Representative Myc, TUNEL, p-βcat, p-eIF2α IF staining. Bar = 100 µm. **B** Quantitation of markers per 400× field from A. Nuclear Myc was scored. The counts in polyps from *APC*^*Min/+*^ mice were included as controls for those in *KI* mice. **C** qRT-PCR analysis of the indicated genes. The relative levels were normalized control polyps in *APC*^*Min/+*^ mice. **D** Representative Ly-6B.2 and CD68 IF staining. **E** Quantitation of cells from (**D**). **B**, **C**, **E**, *n* = 3 mice. ^++^*P* < 0.01, ^+++^*P* < 0.001, ^++++^*P* < 0.0001 (one-way ANOVA and Tukey Post-Hoc test).

To test Myc-dependent mTORi sensitivity in vivo, we took the advantage of the *APC*^*Min/+*^ mice homozygous for the *EIF4E S209A* knockin (4EKI) allele, which strongly inhibits Myc translation and polyposis [19]. The 4EKI polyps had low basal Myc protein and were highly resistant to Everolimus-induced apoptosis (TUNEL), p-eIF2α, or AKT activation (p-βcat) (Fig. 5A, B). 4EKI polyps showed no growth suppression (Ki67), increased p-4EBP1, and no significant changes in *MYC* mRNA on day 3 (Fig. S5B, C). Of note, basal proliferation or apoptosis in the 4EKI polyps was unaffected despite a drastic reduction in Myc and p-4EBP1 (Figs. 5B and S5B). In contrast, 4EKI reduced nuclear Myc+ crypt cells by 50%, which were resistant to Everolimus (Fig. S5D). 4EKI reduced double TUNEL+/p-βcat+ cells in the polyps by over 30-fold (Fig. S5E, F). The induction of cell death-associated ER stress markers (*CHOP, DR5*) and the innate infiltration (neutrophils and macrophages) were blocked in the polyps (Fig. 5C–E). Elevated *ATF3* and *ATF4* indicated induction of ER stress by mTORi. As expected, CD3+ or CD8+ levels did not change obviously in the polyps on day 3, while CD3+ levels were lower in 4EKI *APC*^*Min/+*^ mice (Fig. S5G, H). Together, these findings suggest mTOR and elevated Myc as a vulnerability in mutant *APC*-driven intestinal polyps, and a potential role of cell death in the activation of local immune environment.

### Bid-dependent apoptosis is immunogenic and required for antitumor effects of Everolimus

**Fig. 6:**
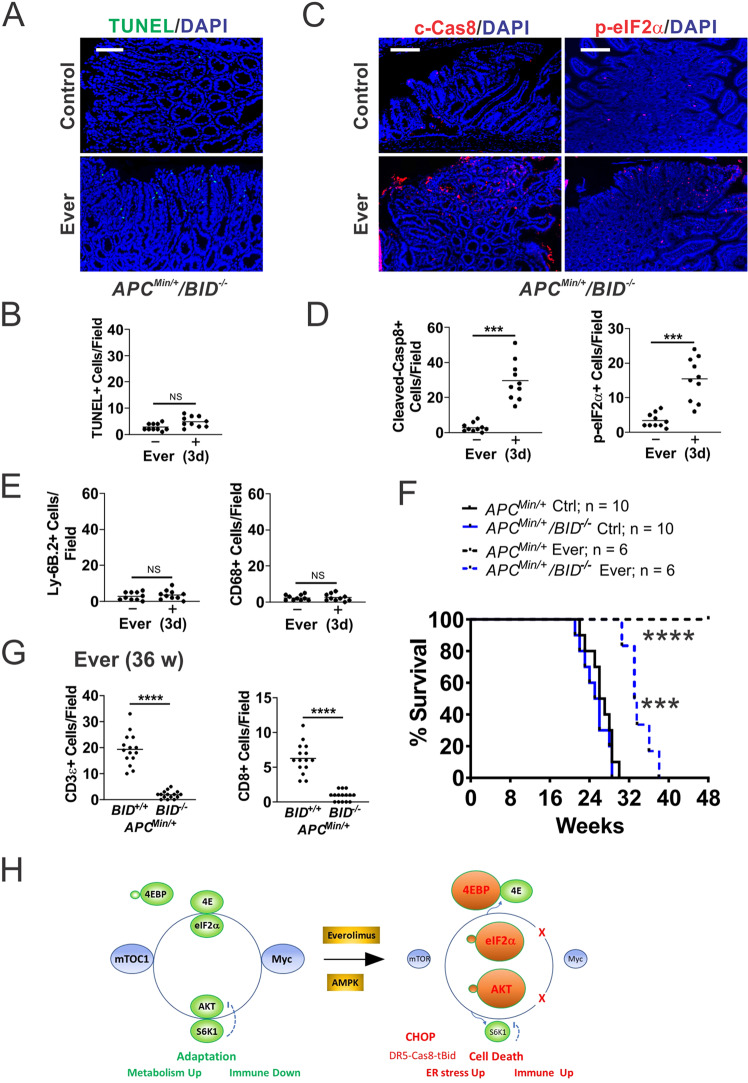
Bid-mediated apoptosis is required for antitumor immunity induced by Everolimus. *APC*^*Min/+*^*/BID*^*−/−*^ mice with established polyps on week 12 were treated with Everolimus for 3 days (**A**–**G**), or till week 48 (48 w). **A** Representative TUNEL IF in the polyps. Bar = 100 µm. **B** Quantitation of TUNEL+ cells in the polyps. **C** Representative c-Cas8 and p-eiF2α IF in the polyps. Bar = 100 µm. **D** Quantitation of indicated cells in the polyps. **E** Quantitation of Ly-6B.2+ cells and CD68+ cells in the polyps. **F** Survival. *APC*^*Min/+*^ mice were included as controls for *BID KO* mice. Log-rank test. ***P < 0.001, *APC*^*Min/+*^*/BID*^*-/-*^ Ctrl *vs*. Ever, ****P < 0.0001 *APC*^*Min/+*^ *vs. APC*^*Min/+*^*/BID*^*−/−*^. **G** Quantitation of CD3+ and CD8+ cells in the polyps of indicated genotypes at 36 w (24 w treatment). **B**, **D**, **E**, **G**, *n* = 3 mice/group. ***P < 0.001, ****P < 0.0001 (Student’s *T*-Test, two-tailed). **H** Working model. Left, elevated Myc and mTOR maintain metabolic and immune adaptation in mutant *APC*-driven polyposis. mTOR inhibition breaks adaptation through Myc-dependent and -independent targets to induce immunogenic cell death required for long-term tumor control. Right, cell death is induced upon inhibition of p-4EBP1 and S6K1, leading to Myc reduction, AKT hyperactivation (p-AKT), ER stress (p-eIF2α/CHOP), and DR5-Cas8-tBid signaling.

If Everolimus-induced cell death is immunogenic, blocking apoptosis, but not stress sensing, should prevent immune recruitment. To test this, we generated *APC*^*Min/+*^/*BID*^*−/−*^ *(KO)* mice and established polyps on week 12 (Fig. 1A). *BID* KO completely blocked everolimus-induced apoptosis in the polyps on day 3 (Fig. 6A, B), but did not affect upstream p-eIF2α induction or caspase-8 cleavage (Fig. 6C, D), or polyp burden as reported (not shown) [23]. *BID* KO led to near complete loss of neutrophil and macrophage infiltration in the polyps (Figs. 6E and S6B), with loss of *CXCL10* induction and loss of suppression of *IL6*, TNFA, *PDL1*, and *PD1* (Fig. S6A). Consequently, the antitumor effect of Everolimus was strongly compromised. All *APC*^*Min/+*^/*BID KO* mice died before week 38, compared to *APC*^*Min/+*^ mice all alive at week 48 (Fig. 6F). Tracking a cohort of mice on week 36, we found elevated CD3+ and CD8+ cells in a few remaining polyps in *APC*^*Min/+*^ mice, similar to week 48 (Fig. S3B). In sharp contrast, *APC*^*Min/+*^*/BID KO* polyps were devoid of T cells (Figs. 6G and S6C). Taken together, these data indicate that ER stress alone is not sufficient, and cell death is required for local immune activation and long-term tumor control.

## Discussion

Mutational inactivation of *APC* is the gateway genetic event in the vast majority of sporadic CRC, and leads to increased Wnt signaling and Myc expression [2]. Elevated Myc in turn drives the proliferation and survival of cancer cells through altered metabolism and enhanced stress adaptation [15]. Genetic ablation or translational suppression of Myc rescues intestinal polyposis in *APC*^*Min/+*^ and conditional APC deficiency mouse models [19, 30–32]. Prior studies reported that rapamycin prevents *APC*-deficiency induced polyposis in several mouse models by inhibiting translation-related targets such as mTORC1, S6 and eEF2 [33–36]. To our knowledge, this study is the first to show that Everolimus treatment not only prevents polyposis, but also clears over 75% of established polyps, and in turn dramatically extends the life of *APC*^*Min/+*^ mice. The efficacy is Myc-dependent, requiring induction of ER stress to activate Bid-dependent apoptosis in the polyps.

Nuclear and cytoplasmic localization of Myc is well-documented, reflecting complex posttranscriptional regulation of Myc activities [12]. Cytoplasmic Myc is observed in cancer or under stress, and can be correlated with poor prognosis [37–39]. Using different antibodies, we detected highly elevated nuclear and cytoplasmic Myc in *APC*-deficient polyps, along with elevated p-4EBP1/p-S6, which is sensitive to mTORi. In contrast, physiologic proliferation and nuclear Myc in the normal crypts are resistant to mTORi. An earlier study reported that ERK activation drives intestinal tumorigenesis in *APC*^*Min/+*^ mice by stabilizing Myc [40]. These findings further support an emerging role of non-transcription and non-nuclear activities of Myc in oncogenesis that cooperate with Myc-dependent transcription. While complete loss of Myc is incompatible with life or impractical in the clinic, it is possible that in Myc-addicted tumors, even modest downregulation of Myc, as we have shown here, is sufficient to impair their survival and adaptation.

mTOR inhibitors including Rapalogs and less selective ATP-competitive agents show little or no single agent activity in most solid tumors including CRC [18, 41]. Mutant *APC*, *KRAS*, and *BRAF* can lead to intrinsic resistance in CRC cells or xenografts [20, 42]. For example, mutant *KRAS* increases Myc translation through p-eIF4E and p-4EBP1, and Everolimus alone was not sufficient to reduce Myc protein or induce ER stress for cell killing [43]. The polyps in *APC*^*Min/+*^ mice have wildtype *KRAS*, Myc expression is primarily dependent on Wnt/β-Catenin signaling and therefore susceptible to mTORi. These findings support deregulated translation as an enabler of oncogenes and oncogenic programs [16, 17], and as a promising target in Myc-driven hematopoietic [44] and intestinal oncogenesis. However, since mTOR has many direct and indirect metabolic and translational targets, targeting Myc in established tumors likely requires combination treatments including agents that can directly block its DNA binding. The peptide Omomyc was the first such agent to enter clinical trials [45].

mTORi treatment induces apoptosis in the polyps through elevated p-eIF2α and ER stress along with TME remodeling. *APC* loss alone led to an increase in Myc, p-eIF2α, and the extrinsic pathway that was further enhanced by Everolimus treatment. Cell death is associated with infiltration of innate cells such as neutrophils and macrophages on day 3 and changes of immune signaling, followed by delayed T-cell infiltration (CD3+ and CD8+) on day 14. Interestingly, ER stress and cell death were preferentially induced in p-βcat (S552)/AKT activated cells, abrogated by *EIF4E S209A* KI or in the normal crypts with low Myc and p-4EBP1. Myc is a well-known inducer of apoptosis in p53 wildtype cells under stress [15]. Myc-dependent apoptosis protects against the clonal expansion of *APC*-deficient cells through immediate killing and establishment of immune surveillance. On the other hand, such a strong immune pressure likely forces adaptation towards a “cold” TME [10, 11]. Rapalogs demonstrate powerful longevity and tumor suppressive benefits in a wide variety of model organisms [18, 46]. It is tempting to speculate the removal of potentially dangerous stem cells with elevated Myc as a key tumor suppressive mechanism.

Our study supports that suppressive immune TME is required to establish Myc-driven polyposis. Drug-induced ICD could be an attractive strategy to re-establish immune-permissive TME and potentially help boost the response to ICBs in more advanced CRCs by reengaging innate immunity to overcome adaptive immune resistance [21, 22, 47]. Elevated p-eIF2α has been proposed as a marker for ICD [21, 48]. We have previously also shown that Non-Steroidal Anti-Inflammatory Drugs (NSAIDs) suppress polyposis in *APC*^*Min/+*^ mice through the induction of ER stress and ICD [23, 24, 49]. *BID* deletion blocks cell death and the innate and adaptive immune response induced by mTORi or Sulindac without affecting ER stress or caspase-8 cleavage. Several NSAIDs such as aspirin, celecoxib, sulindac, and ibuprofen are shown recently to activate AMP-activated protein kinase (AMPK) and inhibit mTOR, independent of COX-2 inhibition [50, 51]. These findings support that mTOR targeting breaks Myc-dependent metabolic and immune adaptation through induction of ER stress and apoptosis mediated by Bid-dependent crosstalk of the extrinsic and intrinsic pathways (Fig. 6H). p-eIF2α defines the core of evolutionally conserved “integrated stress response” (ISR) that signals through numerous targets often coregulated by Myc, ATF4, and CHOP [13, 14]. It would be interesting to determine if activation of a specific ISR kinase and gene signature could predict the effectiveness of Myc targeting and ICD induction.

In summary, our data support a key role of deregulated translation in Myc-driven polyposis and exploitable synthetical lethality of mTOR inhibition and Myc (Fig. 6H). The elevated Myc-ISR axis renders *APC*-deficient cells sensitive to mTOR inhibition via stress hyperactivation and immunogenic cell death. These findings support a key role of Myc in driving CRC development via mTOR-dependent metabolic and immune adaptation, and ICD as powerful TME remodeler and promoter of long-term antitumor immunity. A better understanding of signaling nodes interfacing cell death and local immune modulation in the context of drivers can help develop novel treatments for CRC patients.

## Materials and methods

### Mice and treatment

The procedures for all animal experiments were approved by the Institutional Animal Care and Use Committee of the University of Pittsburgh. All mice were in C57BL/6J background. *APC*^*Min/+*^ mice (Jackson Laboratory) were crossed with *EIF4E S209A* Knockin (*4EKI*) [52] and *BID* Knockout (*BID* KO) [53] mice to generate *APC*^*Min/+*^ mice homozygous for the *4EKI* or *BID* KO alleles. Mouse strains are listed in Table S1. Genotypes were verified by genomic PCR. Genotyping primers are listed in Table S2. Male and female mice were fed AIN-93G diet (Dyets) *ad libitum* starting week 4, and 37.5 mg/kg Everolimus (Sigma) supplementation started at week 4 or 12 as described (Figs. 1A and S1A). For survival studies, mice were followed up to 48 weeks. No animal was censored or removed from the study.

Randomization is used in animal studies as well as analysis. Mice were randomized in Everolimus and control groups. For histology analysis, samples were randomly chosen from three mice in each group, and multiple fields of each mouse or group were quantitated. Total RNA and cDNA were prepared from pooled polyps, 3–5 polyps/mouse, and 3 mice/group. No blinding was used in analysis. Staining markers were confirmed by a second investigator blinded to the group or treatment.

Following sacrifice, intestinal tracts were dissected, rinsed with cold saline, opened longitudinally, and tacked to a foam board for fixation in 10% (vol/vol) formalin. Prior to fixation, macroscopic polyps were removed from some animals for preparation of RNA. After fixation, adenomas were counted under a dissecting microscope, after which tissues were rolled up into “Swiss rolls” for paraffin embedding and histological analysis. Histological analysis was performed by hematoxylin and eosin (H&E) staining.

### Quantitative real-time polymerase chain reaction

For mouse gene expression analysis, freshly dissected polyps were washed in cold PBS, resuspended in 1 ml of TRIzol reagent (Invitrogen), and homogenized in a Dounce homogenizer. Total RNA was then purified according to the manufacturer’s instructions. Polyp total RNA from 3 mice/group was then pooled. For human gene expression analysis, NCM356 cells were harvested, washed once in cold PBS, then lysed in 700 μl RNA lysis buffer. Total RNA was purified using the Quick-RNA MiniPrep kit (Zymo Research) according to the manufacturer’s instructions. cDNA was generated from 2–4 μg of total RNA using SuperScript III reverse transcriptase (Invitrogen) and random primers. Gene expression was quantified with SYBR Green and normalized to *GAPDH* (mouse) or *β-actin* (human). Primer sequences are found in Tables S3 (mouse) and S4 (human).

### Histology and immunostaining

Sections were deparaffinized and rehydrated through graded ethanols. Antigen retrieval was performed by boiling for 10 min in 0.1 M citrate buffer (pH 6.8) with 1 mM EDTA. Non-specific antibody binding was blocked using 20% goat serum (Invitrogen) at room temperature for 1 h unless otherwise indicated. Sections were washed in PBS and incubated overnight at 4 °C in a humidified chamber with diluted primary antibodies for immunohistochemistry (IHC) or immunofluorescence (IF). Quantitation is based on 5–10 fields per group for polyp or “normal” regions per determination. Details on procedures and antibodies are found in supplemental material and Table S5.

### Cell culture and treatment

Normal human colonic cells NCM356 (INCELL) were cultured according to the supplier’s instructions as described [6]. For drug treatment, cells were plated in 12-well plates at ∼30% density 24 h before Everolimus (LC Laboratories) treatment at 10 µM. In some experiments, cells were transfected with small-interfering RNA (siRNA) duplexes with Lipofectamine 2000 (Invitrogen). Details on authentication, treatment, apoptosis and crystal violet staining [19], and transfection with the validated siRNA duplexes targeting *APC*, *BID*, and *MYC* [19, 23] are found in supplemental Table S6.

### Western blotting

Western blotting was performed as previously described [54]. Briefly, cells were harvested at the indicated times after drug treatment, washed once in PBS and lysed in reducing sample buffer. Proteins (30 μg) were separated by SDS–polyacrylamide gel electrophoresis using the NuPAGE system (Invitrogen) and transferred to polyvinylidene difluoride membranes (Immobilon-P, Millipore). Proteins were visualized with ECL (Western Lightning Plus, Perkin Elmer). Representative results are shown, and similar results were obtained in at least three independent experiments. Quantification of western blots was performed with Image J (https://imagej.nih.gov). Each protein was normalized to actin and displayed as a ratio. Details on antibodies are found in Table S5.

### Statistical analysis

Statistical analyses were conducted using GraphPad Prism software (VIII, GraphPad Software Inc., La Jolla, CA). Multiple comparisons were analyzed by one-way analysis of variance (ANOVA) followed by Tukey’s post-hoc test, whereas those between two groups were made by two-tailed, unpaired *t*-test. The means or means ± one standard deviation (s.d.) were displayed in the figures. Survival data were analyzed by log-rank test. Differences were considered significant if the probability of the difference occurring by chance was less than 5 in 100 (*p* < 0.05). Sample size was determined using a combination of published work and power calculations. For ANOVA, we have computed the power for a test of interaction in a two-way factorial design applied by constructing mixed linear growth models to calculate the needed sample size. We estimated that usually three mice per group and five to ten areas total will provide 80% power to detect a standardized interaction of 1.5 SDs as previously described [27, 54].

## Supplementary information

Supplemental text and tables

supplemental figures S1 to S6

## Data Availability

All data needed to evaluate the conclusions in the paper are present in the paper and/or the Supplementary Materials.
