# Supplementary material for: mTOR inhibition suppresses Myc-driven polyposis by inducing immunogenic cell death
Source: Oncogene. Author manuscript; Available in PMC 2023 Jul 10. (PMC10256613; doi:10.1038/s41388-023-02706-6)
Supplement: supplemental text [file NIHMS1898478-supplement-supplemental_text.docx]

**mTOR inhibition suppresses Myc-driven polyposis by**

**inducing immunogenic cell death**

Brian J. Leibowitz ^1,2^, Guangyi Zhao ^1^, Wenxin Xia ^1,$^, Yuhan Wang ^1,*^, Hang Ruan ^1^, Lin Zhang^3,*^, and Jian Yu^1, 2,*^

^1^Department of Pathology, ^2^Department of Radiation Oncology, ^3^Department of Pharmacology and Chemical Biology, University of Pittsburgh School of Medicine, UPMC Hillman Cancer Center, Pittsburgh, PA 15213.

^$^Current affiliation: Department of Biochemistry and Molecular Pharmacology at New York University Grossman School of Medicine, New York, NY 10016.

^*^Current affiliation: Department of Medicine, University of Southern California, Keck School of Medicine, Los Angeles, CA 90033.

**Correspondence:**

Jian Yu, Ph.D. USC Keck School of Medicine, 1450 Biggy Street, NRT Rm 4501, Los Angeles, CA90033. Email: [yujian@usc.edu](mailto:yujian@usc.edu), or jiy3@pitt.edu.

**Running head:** **Synthetic lethality of mTORi and Myc**

**Key words: mTOR, Myc, ER stress, colon cancer, polyposis**

**Supplemental Materials & Methods**

**Histology and Immunostaining**

Sections were deparaffinized and rehydrated through graded ethanols. Antigen retrieval was performed by boiling for 10 minutes in 0.1 M citrate buffer (pH 6.8) with 1 mM EDTA. Non-specific antibody binding was blocked using 20% goat serum (Invitrogen) at room temperature for 1 h unless otherwise indicated. Sections were washed in PBS and incubated overnight at 4^o^C in a humidified chamber with diluted primary antibodies for immunohistochemistry (IHC) or immunofluorescence (IF). Quantitation is based on 5-10 fields per group for polyp or “normal” regions per determination. Details on procedures and antibodies are found in supplemental material and Table S5.

*Ki67*: Following primary antibody incubation, sections were incubated with AlexaFluor 594-conjugated goat anti-rat secondary antibodies (1:400) for 1 hour at room temperature. Sections were then washed in PBS and mounted with VectaShield + DAPI (Vector Labs). For IHC, following deparaffinization and rehydration, sections were incubated in 3% H2O2 for 5 minutes, washed in PBS, and boiled for 10 minutes in citrate buffer as described. Sections were then blocked in 20% goat serum for 1 h and incubated overnight in diluted primary antibody. Following primary antibody incubation, sections were washed in PBS and incubated with biotin-conjugated goat anti-rat secondary antibodies (1:100) for 1 hour at room temperature. Sections were then washed in PBS and incubated with biotin-avidin (ABC Reagent, Vector Labs) for 30 minutes at room temperature. Slides when then passed in PBS, developed with DAB reagent (Vector Labs), counterstained with hematoxylin, and mounted with Permount (Fisher).

*Phospho-4EBP1*: Following primary antibody incubation, sections were then incubated with AlexaFluor 594-conjugated goat anti-rabbit secondary antibodies (1:400) for 1 hour at room temperature. Sections were then washed in PBS and mounted with VectaShield + DAPI (Vector Labs) for visualization.

*Phospho-S6*: Following primary antibody incubation, sections were then incubated with AlexaFluor 594-conjugated goat anti-rabbit secondary antibodies (1:400) for 1 hour at room temperature. Sections were then washed in PBS and mounted with VectaShield + DAPI (Vector Labs).

*Myc*: Following primary antibody incubation, sections were then incubated with AlexaFluor 594-conjugated goat anti-rabbit secondary antibodies (1:400) for 1 hour at room temperature. Sections were then washed in PBS and mounted with VectaShield + DAPI (Vector Labs). Two different antibodies were used.

*Phospho-eIF2α*: Following primary antibody incubation, sections were then incubated with AlexaFluor594-conjugated goat anti-rabbit secondary antibodies (1:400) for 1 hour at room temperature. Sections were then washed in PBS and mounted with VectaShield + DAPI (Vector Labs).

*Cleaved Caspase-8*: Following primary antibody incubation, sections were then incubated with AlexaFluor594-conjugated goat anti-rabbit secondary antibodies (1:400) for 1 hour at room temperature. Sections were then washed in PBS and mounted with VectaShield + DAPI (Vector Labs).

TUNEL: Cell death was measured using the ApopTag Fluorescein In Situ Apoptosis Detection Kit (Chemicon) according to the manufacturer’s instructions.

p-β-catenin (S552) IF. Following primary antibody incubation (1:100) with rabbit-anti-p-β-catenin (gift from Linheng Li). Sections were incubated with AlexaFluor-594 goat-anti-Rabbit secondary antibodies (1:400) for 1 hour at room temperature. Sections were then washed in PBS and mounted with VectaShield plus DAPI (Vector Labs).

p-βcat (S552)/TUNEL double IF. Sections were stained for p-βcat (S552) as above. After washing in PBS, TUNEL staining was performed according to the manufacturer’s instructions

*CD3ε*: Following primary antibody incubation, sections were then incubated with AlexaFluor594-conjugated goat anti-rabbit secondary antibodies (1:400) for 1 hour at room temperature. Sections were then washed in PBS and mounted with VectaShield + DAPI (Vector Labs).

*CD8*: Prior to primary antibody incubation, sections were incubated with non-immune rabbit IgG (1:300) overnight at 4^o^C. Sections were then washed in PBS and incubated overnight with the primary antibody, followed by incubation with biotin-conjugated goat anti-mouse secondary antibodies (1:100) for 1 hour at room temperature, washing and incubation for 30 minutes with AlexaFluor594-conjugated streptavidin (1:400). Sections were then washed in PBS and mounted with VectaShield + DAPI (Vector Labs).

*Ly-6B.2*: Prior to primary antibody incubation, sections were incubated with non-immune rabbit IgG (1:300) overnight at 4^o^C. Sections were then washed in PBS and incubated overnight with the primary antibody, followed by incubation with biotin-conjugated goat anti-rat secondary antibodies (1:100) for 1 hour at room temperature, followed by washing and incubation for 30 minutes with AlexaFluor594-conjugated streptavidin (1:400). Sections were then washed in PBS and mounted with VectaShield + DAPI (Vector Labs).

*CD68*: Prior to primary antibody incubation, sections were incubated with non-immune rabbit IgG (1:300) overnight at 4^o^C. Sections were then washed in PBS and incubated overnight with the primary antibody, followed by incubation with biotin-conjugated goat anti-mouse secondary antibodies (1:100) for 1 hour at room temperature, followed by washing and incubation for 30 minutes with AlexaFluor594-conjugated streptavidin (1:400). Sections were then washed in PBS and mounted with VectaShield + DAPI (Vector Labs).

**Cell Culture and Treatment**

Normal human colonic cells NCM356 (INCELL) are cultured according to the supplier’s instructions. Cells were maintained at 37 °C and 5% CO2 and in cultured in M3 media (INCELL) supplemented with 10% (vol/vol) defined FBS (R&D), 100 units/mL penicillin, and 100 μg/mL streptomycin (Invitrogen). Cell lines in culture were regularly monitored for the absence of *Myc*oplasma, genotype and morphology approximately every 6 months. Cell lines are generally used for less than 2 months or fewer than 12 passages upon thawing from LN tank. For drug treatment, cells were plated in 12-well plates at ∼30% density 24 h before Everolimus (LC Laboratories) treatment at 10 µM.

Attached and floating cells were harvested at the indicated times after drug treatment and analyzed for apoptosis by counting cells with condensed chromatin and micronucleation after nuclear staining with Hoechst 33258.

Crystal violet staining. Following treatment, attached cells were stained with crystal violet (Sigma, St. Louis, MO, Cat# C0775) (3.7% Paraformaldehyde, 0.05% crystal violet in distilled water) as described. Each assay was performed in triplicate with representative images shown.

In some experiments, cells were transfected with small-interfering RNA (siRNA) duplexes with Lipofectamine 2000 (Invitrogen). Briefly, 200–400 pmols of siRNA was transfected into cells in 12-well plates for 4 h, followed by incubation in medium containing 5% FBS for 20 h prior to drug treatment. Details on the validated siRNA duplexes targeting *APC*, *BID*, and *MYC*  are found in Table S6.

**Supplemental Tables**

| **Table S1: Mouse strains used in this study** | | | |
| --- | --- | --- | --- |
| **Strain Name** | **Common Name** | **Source** | **PMID** |
| C57BL/6J, *Apc^Min^* | APCMin/+ | Jackson Labs | 1350108 |
| C57BL/6J, BID-/- | BID KO | XM Yin | 10476969 |
| C57BL/6, EIF4E S209A | eIF4E KI | N. Sonenberg | 20679199 |

| **Table S2: Genotyping Primers used in this study** | | |
| --- | --- | --- |
| **Gene** | **Primer** | **Sequence** |
| APCMin/+ | Wild Type | 5'-GCCATCCCTTCACGTTAG |
| APCMin/+ | Common | 5'-TTCCACTTTGGCATAAGGC |
| APCMin/+ | Mutant | 5'-TTCTGAGAAAGACAGAAGTTA |
| 4ES209A | Forward | 5'-TTTGAAATTGGTTTGTAAAGTTGG |
| 4ES209A | Reverse | 5'-GCAATGCAAGTCGAAATGTG |
| BID KO | Wild Type | 5'-GAGATGGACCACAACATC |
| BID KO | Common | 5'-CCGAAATGTCCCATAAGAG |
| BID KO | Mutant | 5'-TGCTACTTCCATTTGTCACGTCCT |

| **Table S3: Mouse qRT-PCR Primers used in this study** | | | |
| --- | --- | --- | --- |
| **Gene** | **Primer** | **Sequence** | **Function** |
| DDIT3 (CHOP) | Forward | 5'-CTGCCTTTCACCTTGGAGAC | ER Stress |
| DDIT3 (CHOP) | Reverse | 5'-CGTTTCCTGGGGATGAGATA |  |
| ATF3 | Forward | 5'-AACTGGCTTCCTGTGCACTT | ER Stress |
| ATF3 | Reverse | 5'-TGAGGCCAGCTAGGTCATCT |  |
| ATF4 | Forward | 5'-GAGCTTCCTGAACAGCGAAGTG | ER Stress |
| ATF4 | Reverse | 5'-TGGCCACCTCCAGATAGTCATC |  |
| TNFRSF10b (DR5) | Forward | 5'-AAAACGGCTTGGGCATCTTGGC | Cell Death |
| TNFRSF10b (DR5) | Reverse | 5'-AGACGGTTCCAGGAGTCAAAGG |  |
| IL6 | Forward | 5’-AGGATACCACTCCCAACAGACCT | Cytokine |
| IL6 | Reverse | 5’-CAAGTGCATCATCGTTGTTCATAC |  |
| TNFA | Forward | 5’-TTCTGTCTACTGAACTTCGGGGTGATCGGTCC | Cytokine |
| TNFA | Reverse | 5’-GTATGAGATAGCAAATCGGCTGACGGTGTGGG |  |
| MYC | Forward | 5′-TCTCCACTCACCAGCACAACTACG | Wnt |
| MYC | Reverse | 5′-ATCTGCTTCAGGACCCT |  |
| CXCL10 | Forward | 5'-CCAAGTGCTGCCGTCATTTTC | M1 |
| CXCL10 | Reverse | 5'-GGCTCGCAGGGATGATTTCAA |  |
| NOS2 | Forward | 5'-GTTCTCAGCCCAACAATACAAGA | M1 |
| NOS2 | Reverse | 5'-GTGGACGGGTCGATGTCAC |  |
| TREM2 | Forward | 5'-CTGGAACCGTCACCATCACTC | M2 |
| TREM2 | Reverse | 5'-CGAAACTCGATGACTCCTCGG |  |
| ARG1 | Forward | 5'-TGGCTTGCGAGACGTAGAC | M2 |
| ARG1 | Reverse | 5'-GCTCAGGTGAATCGGCCTTTT |  |
| CD4 | Forward | 5'-AGGTGATGGGACCTACCTCTC | T Cell |
| CD4 | Reverse | 5'-GGGGCCACCACTTGAACTAC |  |
| CD8 | Forward | 5'-CCGTTGACCCGCTTTCTGT | T Cell |
| CD8 | Reverse | 5'-CGGCGTCCATTTTCTTTGGAA |  |
| PD1 | Forward | 5'-TGCTCAACAAGTATGTCAGAGG | checkpoint |
| PD1 | Reverse | 5'-ACACTAGGGACAGGTGCTGC |  |
| PDL1 | Forward | 5'-TCAGCTACGGTGGTGCGGACT | checkpoint |
| PDL1 | Reverse | 5'-AGCTTCTGGATAACCCTCGGCCT |  |
| GAPDH | Forward | 5´-CTCTGGAAAGCTGTGGCGTGATG | control |
| GAPDH | Reverse | 5´-ATGCCAGTGAGCTTCCCGTTCAG |  |

| **Table S5: Antibodies used in this study** | | | | | |
| --- | --- | --- | --- | --- | --- |
| **Antigen** | **Species** | **Application** | **Dilution** | **Vendor** | **Catalog** |
| Ki67 | rat | IHC/IF | 1/100 | DAKO | M7249 |
| p-4EBP1 (S65/70) | rabbit | WB, IF | 1/1000, 1/100 | Cell Signaling | 9451 |
| p-S6 (235/236) | rabbit | IF | 1/100 | Cell Signaling | 2211 |
| p-Akt (S473) | rabbit | WB, IF | 1/1000, 1/100 | Cell Signaling | 9271 |
| Myc | rabbit | IF | 1/100 | Cell Signaling | 13987 |
| Myc | rabbit | IF | 1/100 | Abcam | 39688 |
| p-eIF2α (S51) | rabbit | WB, IF | 1/1000, 1/100 | Cell Signaling | 3398 |
| p-β-catenin (S552) | rabbit | IF | 1/100 | Linheng Li | NA |
| cleaved caspase 8 | rabbit | IF | 1/600 | Novus Biologicals | NB100-56116 |
| CD3 | rabbit | IF | 1/50 | Fisher | RB-360-A0 |
| CD8 | mouse | IF | 1/50 | Fisher | 14-0195-82 |
| Ly-6B.2 | rat | IF | 1/100 | Bio-Rad | MCA771GT |
| CD68 | mouse | IF | 1/100 | Abcam | ab955 |
| Normal Rabbit IgG | rabbit | IF | 1/300 | R&D Systems | AB-105-C |
| Anti-Rabbit Alexa-594 | goat | IF | 1/400 | Invitrogen | A11012 |
| Anti-Rat Alexa-594 | goat | IF | 1/400 | Invitrogen | A-11007 |
| Anti-Rat Biotin | goat | IF | 1/100 | Pierce | 31830 |
| Anti-Rabbit Biotin | goat | IF | 1/100 | Pierce | 31822 |
| Anti-Mouse Biotin | goat | IF | 1/100 | Pierce | 31802 |
| Streptavidin Alexa-594 | N/A | IF | 1/400 | Invitrogen | S-11227 |
| p-eIF4E (S209) | rabbit | WB | 1/1000 | Cell Signaling | 9741 |
| Myc (human) | mouse | WB | 1/500 | Santa Cruz | SC-40 (9E10) |
| BID | rabbit | WB | 1/1000 | Cell Signaling | 2002 |
| caspase 8 | mouse | WB | 1/1000 | Cell Signaling | 9746 |
| Caspase 3 (cleaved, Asp 175) | rabbit | WB | 1/1000 | Cell Signaling | 9661 |
| β-actin | mouse | WB | 1/5000 | Sigma | A5441 |

| **Table S6: siRNA Sequences used in this study** | |
| --- | --- |
| Gene | Sequence |
| APC-157 | 5′-GGAAGUAUUGAAGAUGAAG |
| BID-238 | 5′-GAAGACAUCAUCCGGAAUA |
| cMyc-55 | 5′-AACGUUAGCUUCACCAACAUU |

**Supplementary Figure Legends**

**Figure S1.** **Everolimus prevents polyp formation in *APC*^Min/+^ mice**. **(A)** *APC*^Min/+^ mice were fed control AIN-93G diet (Ctrl) with or without Everolimus at weaning (week 4). Tumor burden was analyzed at 12 weeks (12 w), and survival was analyzed at 48 weeks. (**B**) Kaplan-Meier curve. Log-Rank test. (**C**) Quantitation of macroscopic adenomas at week 12. (**D**) Representative images of small intestine and colon at week 12. Bar = 1 cm. (**E**) Higher magnification images from D. Bar = 1 cm. C, values are mean +/- SEM, n = 3 mice/group. **** *P* < 0.01 (Student’s T-Test, two-tailed).

**Figure S2.** **Everolimus has little impact on the “normal” intestinal crypts**. *APC*^Min/+^ mice with established polyps were treated with Everolimus for 3 days***.***  **(A)** Representative Ki67 IHC in non-polyp (“normal”) regions. (**B**) Representative IF of the indicated markers in non-polyp regions. Bars = 100 μm. (**C**) Quantitation of indicated markers from B. Nuclear Myc was scored. (**D**) Representative IF of stress and death markers in non-polyp regions. Bars = 100 μm. (**E**) Quantitation of indicated markers from D. **(F)** Representative Myc IF images with predominant cytoplasmic signals detected by a polyclonal rabbit antibody in the polyps and non-polyp regions. Bars = 100 μm. Higher magnification images are shown in insets. **(G)** Quantitation of Myc IF from F. **(H)** Higher magnification Myc IF images in the polyps from Fig. 1H with predominant nuclear signals. C, E, G, values are mean +/- SEM, n = 3 mice/group. **P* < 0.05, ** *P* < 0.01 (Student’s T-Test, two-tailed).

**Figure S3. Analysis of immune markers in the polyps**. *APC*^Min/+^ mice with established polyps were treated with Everolimus for 3 days (3d) or until week 48. **(A)** qRT-PCR analysis of the indicated immune markers in polyps at 3d. Values are mean +/- SEM, n = 3 mice/group. **P* < 0.05, ** *P* < 0.01 (Student’s T-Test, two-tailed). (**B**) Representative CD8, Ly-6B.2 and CD68 IHC staining in residual polyps at 48 w.

**Figure S4. The effect of *APC* knockdown in normal human colonic cells after Everolimus treatment. (A)** siRNA knockdown (KD) of *APC* or *MYC* **(B)** in human NCM356 cells was examined by western blot at 24 hours. Actin is the loading control. **(C)** NCM-356 cells transfected with the indicated siRNA were treated +/- Everolimus 10 µM for 24 h. qRT-PCR analysis of the indicated genes. Values are means +/- SEM, n = 3 mice/group. **P* < 0.05 (Student’s T-Test, two-tailed). **(D)** Quantification of western blots by Image J. Each protein was normalized to actin and displayed as ratio. KD efficiency was calculated to that of the control siRNA (CT). BID was based on lane 3 and 5 in Fig. 4H.

**Figure S5. *EIF4ES209A* KI markedly reduces Myc protein and blocks everolimus-induced stress in polyps.** *APC^Min/+^* and *APC^Min/+^/4EKI* mice with established polyps at 12 weeks were treated with Everolimus for three days (3d). (**A**) Representative p-4EBP1 and Ki67 IF images in polyps from *APC^Min/+^/4EKI* mice. Bar = 100 µm.  **(B**) Quantification of cells per 400X field from A in the indicated genotypes. (**C)** qRT-PCR analysis of *MYC* in the polyps. (**D)** Quantification of nuclear Myc+ cells in non-polyp regions. Right, representative Myc IF in *APC^Min/+^/4EKI* crypts Bars = 100 μm. **(E)** Representative TUNEL/p-βcat double IF staining in the polyps. Bar = 100 µm. Higher magnification images including single channel are shown in insets. **(F)** Quantification TUNEL/p-βcat double IF in indicated genotypes. **(G)** Representative CD3ε and CD8 IF staining in the polyps**. (H**) Quantitation of cells per 400X field from G. B, C, D, F, H are from n = 3 mice/group. 3-4 randomly chosen polyps/mouse. C, cDNA was made from pooled polyps. *****P* < 0.0001 (Student’s T-Test, two-tailed). ^+^*P* < 0.05, ^++^*P* < 0.01, ^+++^*P* < 0.001, ^++++^*P* < 0.0001 (One-Way ANOVA and Tukey Post-Hoc test).

**Figure S6.** **Bid-dependent apoptosis is required for Everolimus-induced anti-tumor immunity.** *APC*^Min/+^/BID^-/-^ mice with established polyps were treated with Everolimus. The intestine and polyps were analyzed at indicated time points. (**A**) qRT-PCR analysis of the indicated stress genes in polyps at day 3. Values are mean +/- SEM, n = 3 mice/group. cDNA was made from pooled polyps. **P* < 0.05, ***P* < 0.01 (Student’s T-Test, two-tailed). **(B)** Representative Ly-6B.2 and CD68 IF staining in polyps at day 3. Bar = 100 µm (**C**) Representative CD3ε and CD8 IF staining in *APC^Min/+^* and *APC^Min/+^/BID-/-* mice at 36 w (24 w treatment). Bar = 100 µm.
