# Supplementary material for: mTOR inhibition suppresses Myc-driven polyposis by inducing immunogenic cell death
Source: Oncogene. Author manuscript; Available in PMC 2023 Jul 10. (PMC10256613; doi:10.1038/s41388-023-02706-6)
Supplement: supplemental figures [file NIHMS1898478-supplement-supplemental_figures.pdf]

Figure S1

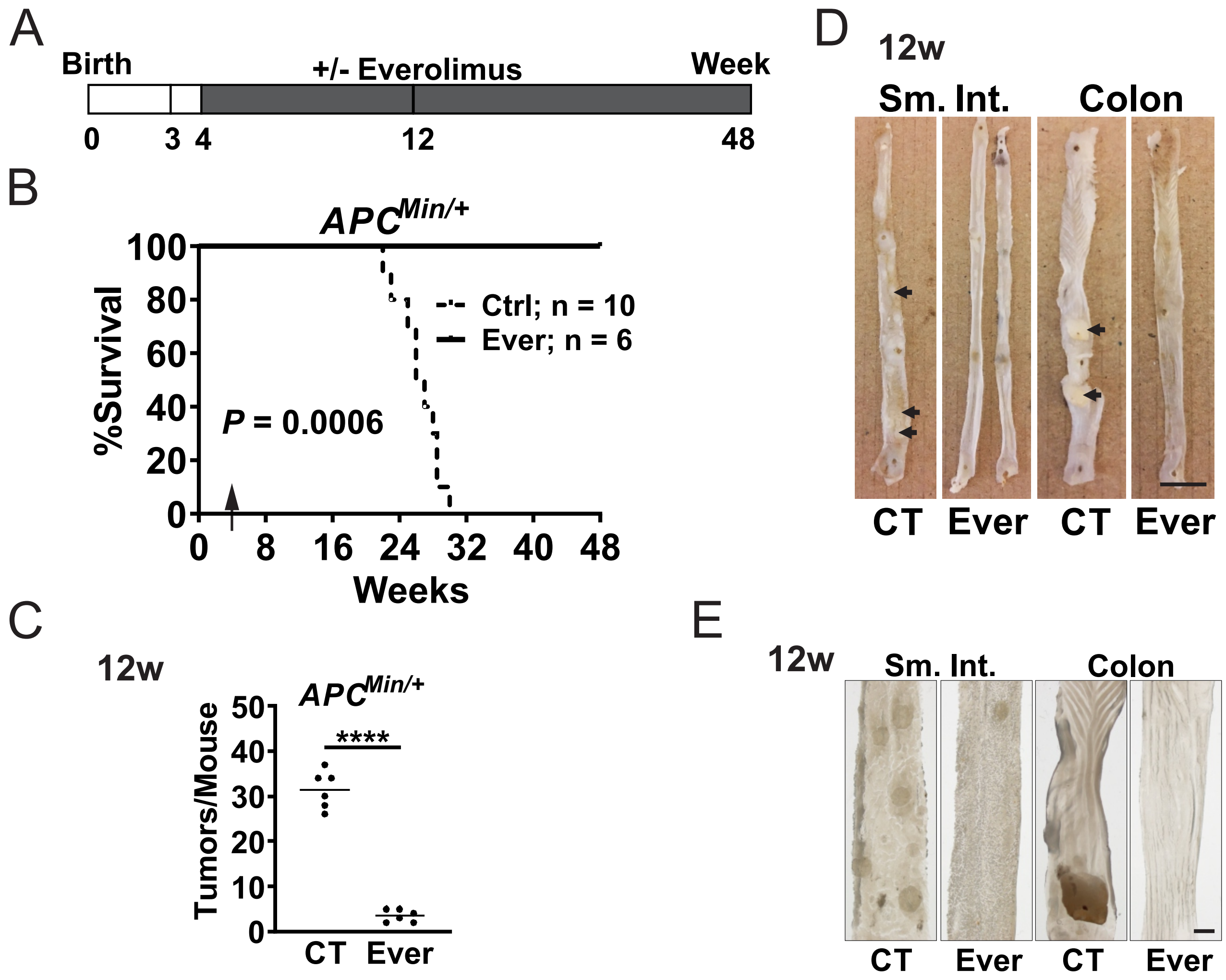

A
Birth
+/- Everolimus
Week
0
3
4
12
48
B
APCMin/+
100
80
60
40
20
0
%Survival
Ctrl; n = 10
Ever; n = 6
P = 0.0006
0
8
16
24
32
40
48
Weeks
C
12w
APCMin/+
50
40
30
20
10
0
Tumors/Mouse
****
CT
Ever
D
12w
Sm. Int.
Colon
CT
Ever
CT
Ever
E
12w
Sm. Int.
Colon
CT
Ever
CT
Ever

Figure S2

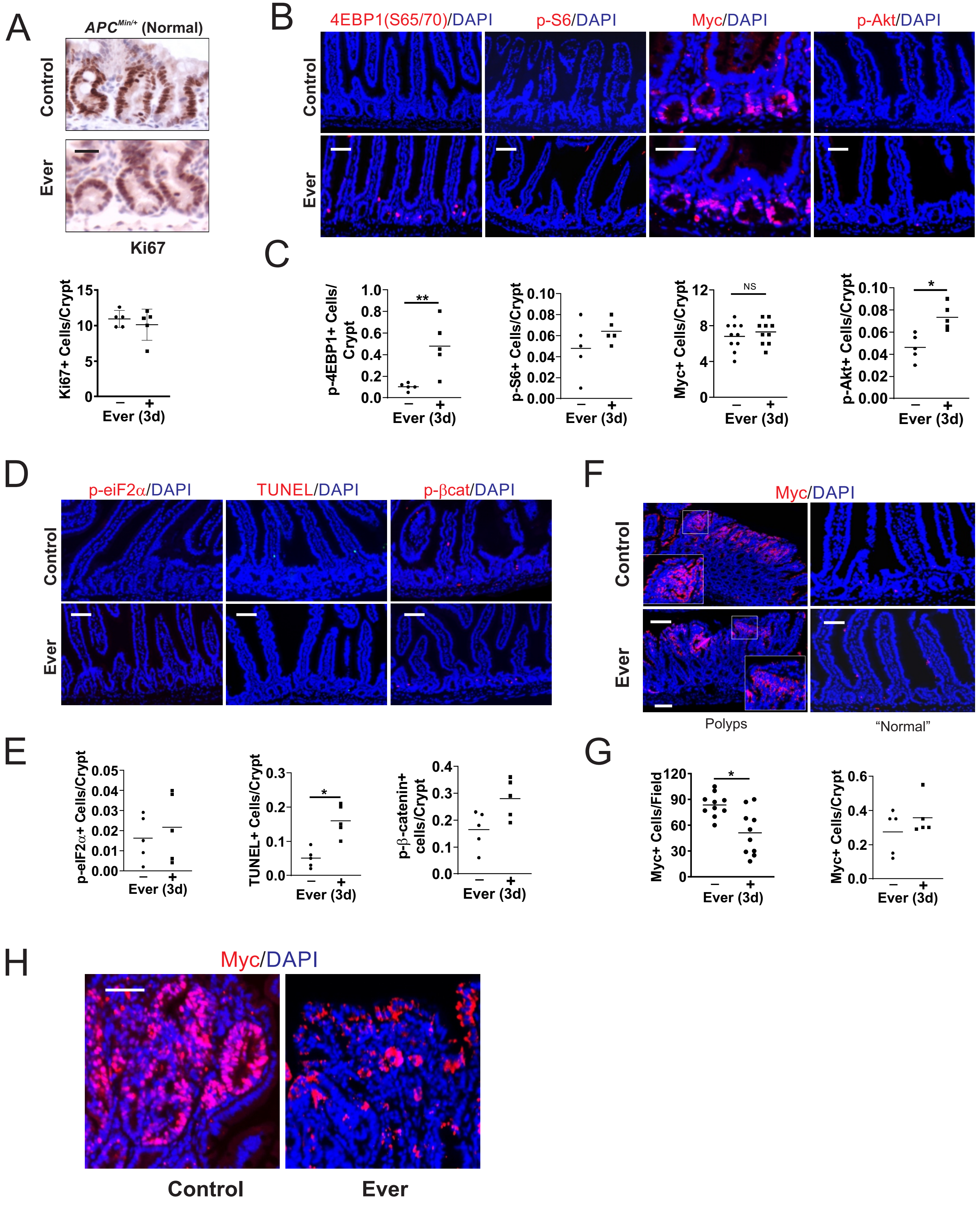

A
$APC^{Min/+}$ (Normal)
Control
Ever
Ki67
Ki67+ Cells/Crypt
Ever (3d)
B
4EBP1(S65/70)/DAPI
p-S6/DAPI
Myc/DAPI
p-Akt/DAPI
Control
Ever
C
p-4EBP1+ Cells/Crypt
p-S6+ Cells/Crypt
Myc+ Cells/Crypt
p-Akt+ Cells/Crypt
NS
Ever (3d)
D
p-eiF2α/DAPI
TUNEL/DAPI
p-βcat/DAPI
Control
Ever
E
p-eIF2α+ Cells/Crypt
TUNEL+ Cells/Crypt
p-β-catenin+ cells/Crypt
Ever (3d)
F
Myc/DAPI
Control
Ever
Polyps
"Normal"
G
Myc+ Cells/Field
Myc+ Cells/Crypt
Ever (3d)
H
Myc/DAPI
Control
Ever

Figure S3

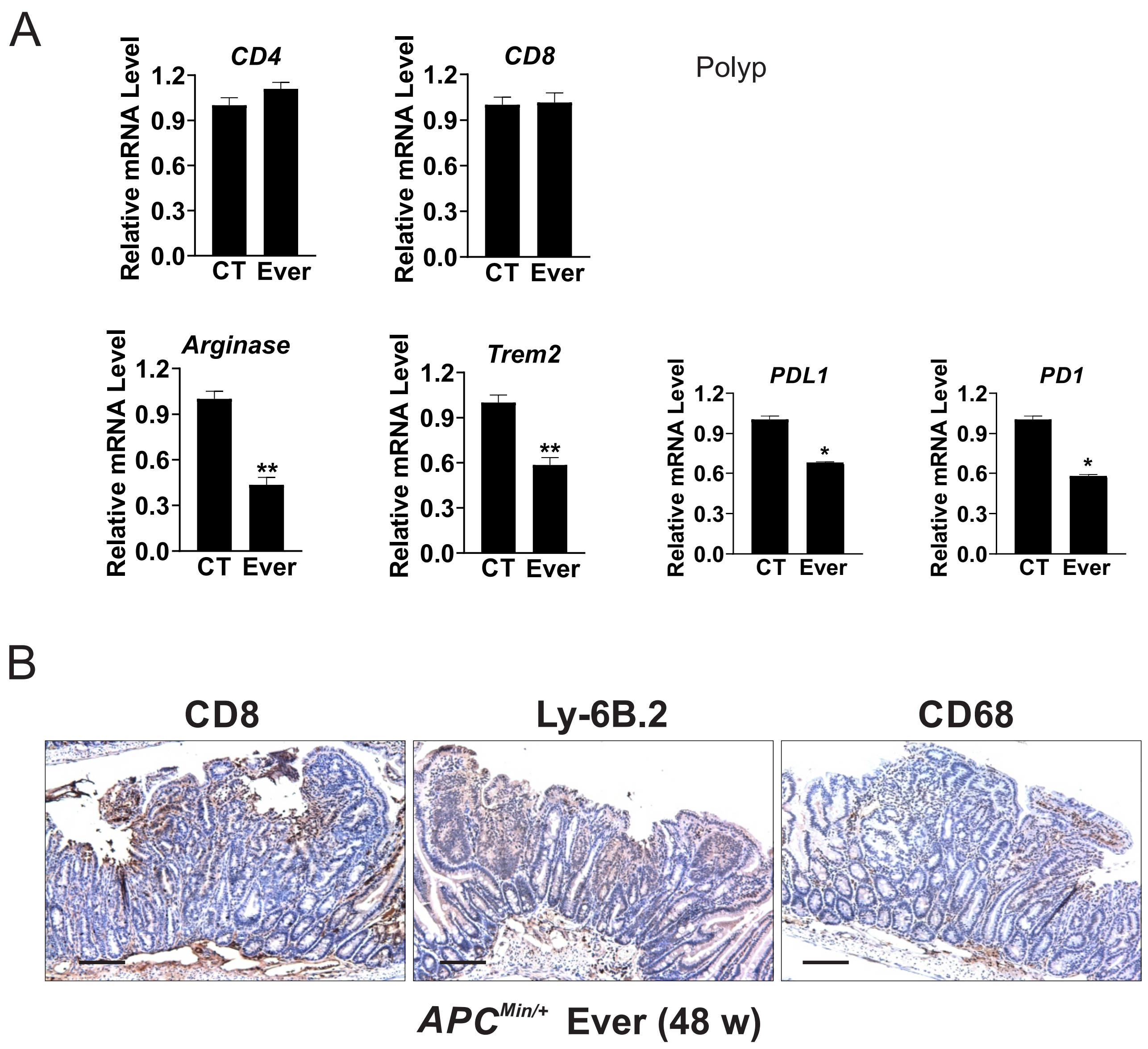

A
CD4
CD8
Polyp
Arginase
Trem2
PDL1
PD1
Relative mRNA Level
1.2
0.9
0.6
0.3
0.0
CT
Ever
**
*
B
CD8
Ly-6B.2
CD68
APC^Min/+ Ever (48 w)

Figure S4

A

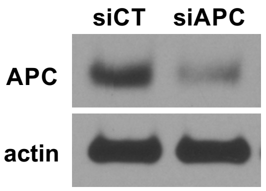

B

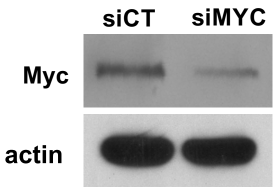

C

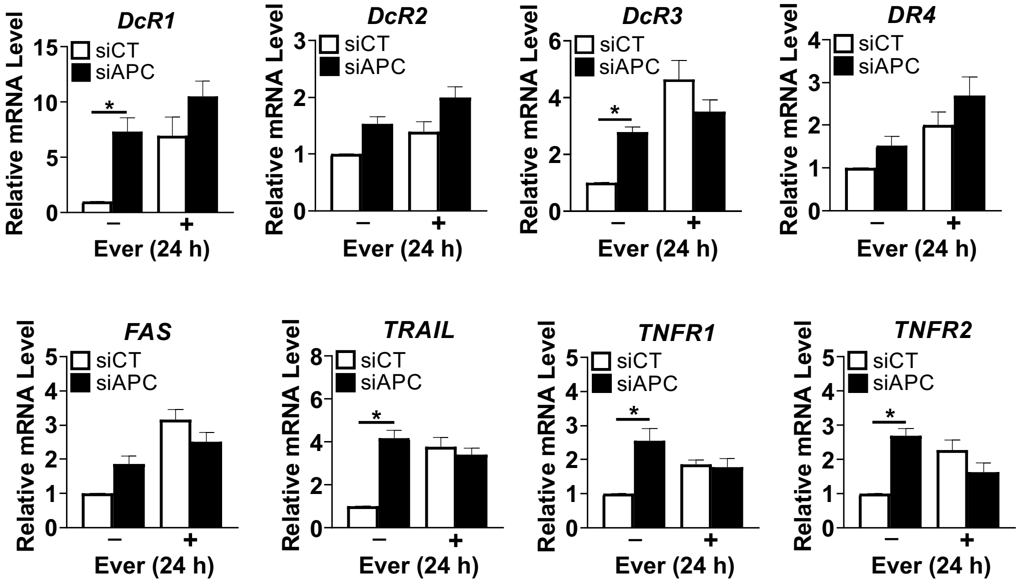

D

4D	Un	Ever	un	Ever
p-4EBP1	0.99	0.21	0.95	0.30
p-S6	0.73	0.15	0.75	0.17
p-AKT	0.11	0.90	0.22	0.43
p-eIF4E	0.39	0.36	0.61	0.70
Myc	0.11	0.11	0.18	0.24
p-eIF2a	0.09	0.44	0.24	0.38

4E	Un	Ever	Un	Ever	Un	Ever
SiRNA	CT	CT	APC	APC	APC/MYC	APC/MYC
Cas8_p18	0.17	0.28	0.35	0.96	0.23	0.37
tBid	0.17	0.13	0.37	0.59	0.26	0.15
*Cas3	0.18	0.17	0.35	0.74	0.11	0.14

4H	Un	Ever	un	Ever	Un	Ever
SiRNA	CT	CT	APC	APC	APC/BID	APC/BID
Cas8_p18	0.03	0.04	0.14	0.48	0.07	0.16
tBid	0.03	0.04	0.08	0.67	0.03	0.01
*Cas3	0.03	0.04	0.12	0.52	0.05	0.14

S4A,B/4H						
sIRNA	CT	APC	CT	MYC	CT	BID
Ratio (actin)	0.90	0.35	0.51	0.17	3.35	0.66
% vs CT	100%	39%	100%	34%	100%	20%
KD (%)	N/A	61%	N/A	66%	N/A	80%

Figure S5

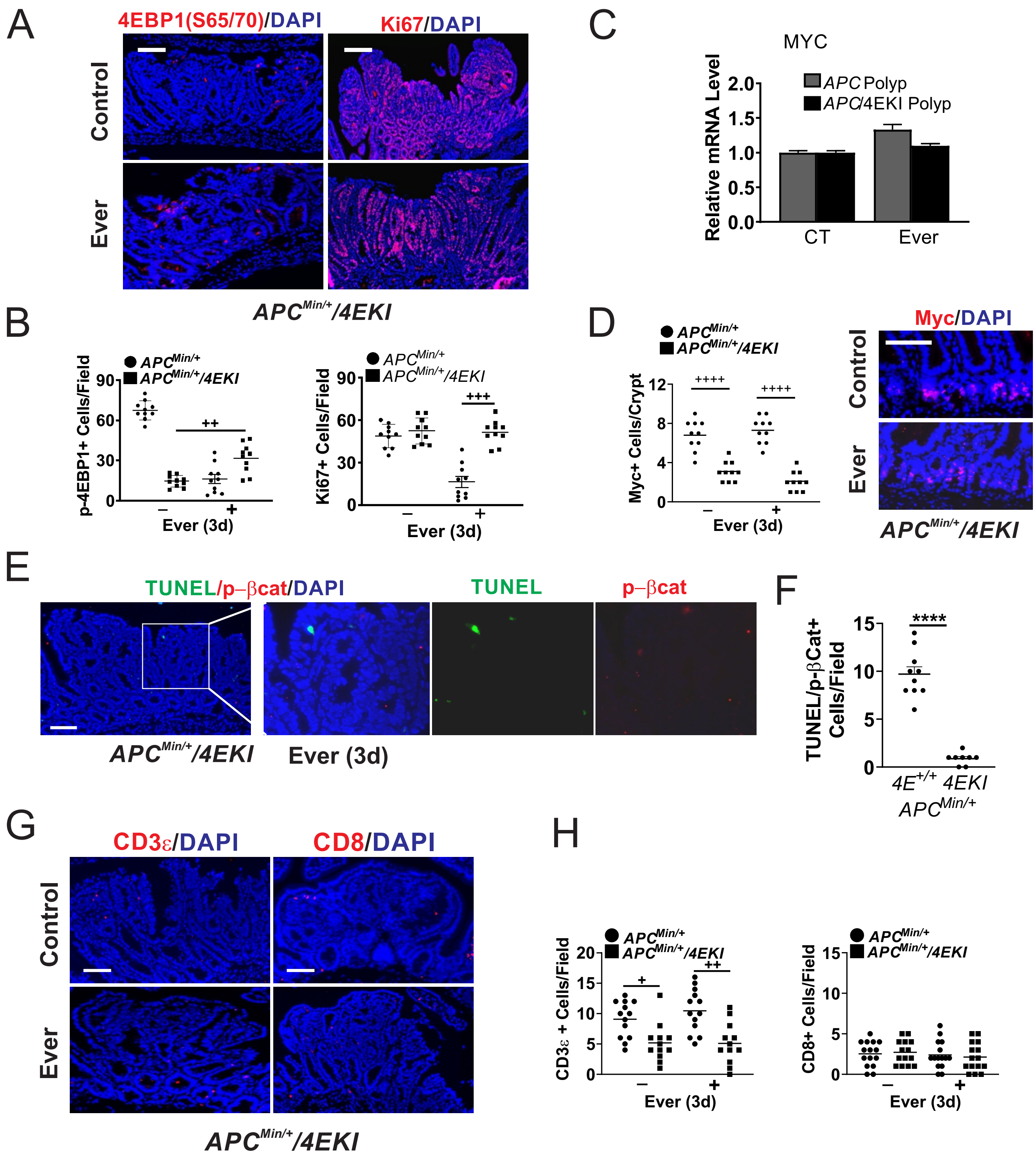
A
4EBP1(S65/70)/DAPI
Ki67/DAPI
Control
Ever
APCMin/+/4EKI
B
p-4EBP1+ Cells/Field
APCMin/+
APCMin/+/4EKI
++
Ever (3d)
Ki67+ Cells/Field
+++
C
MYC
Relative mRNA Level
APC Polyp
APC/4EKI Polyp
CT
Ever
D
Myc+ Cells/Crypt
++++
Myc/DAPI
E
TUNEL/p-βcat/DAPI
TUNEL
p-βcat
Ever (3d)
F
TUNEL/p-βCat+ Cells/Field
****
4E+/+
4EKI
APCMin/+
G
CD3ε/DAPI
CD8/DAPI
H
CD3ε + Cells/Field
+
CD8+ Cells/Field

Figure S6

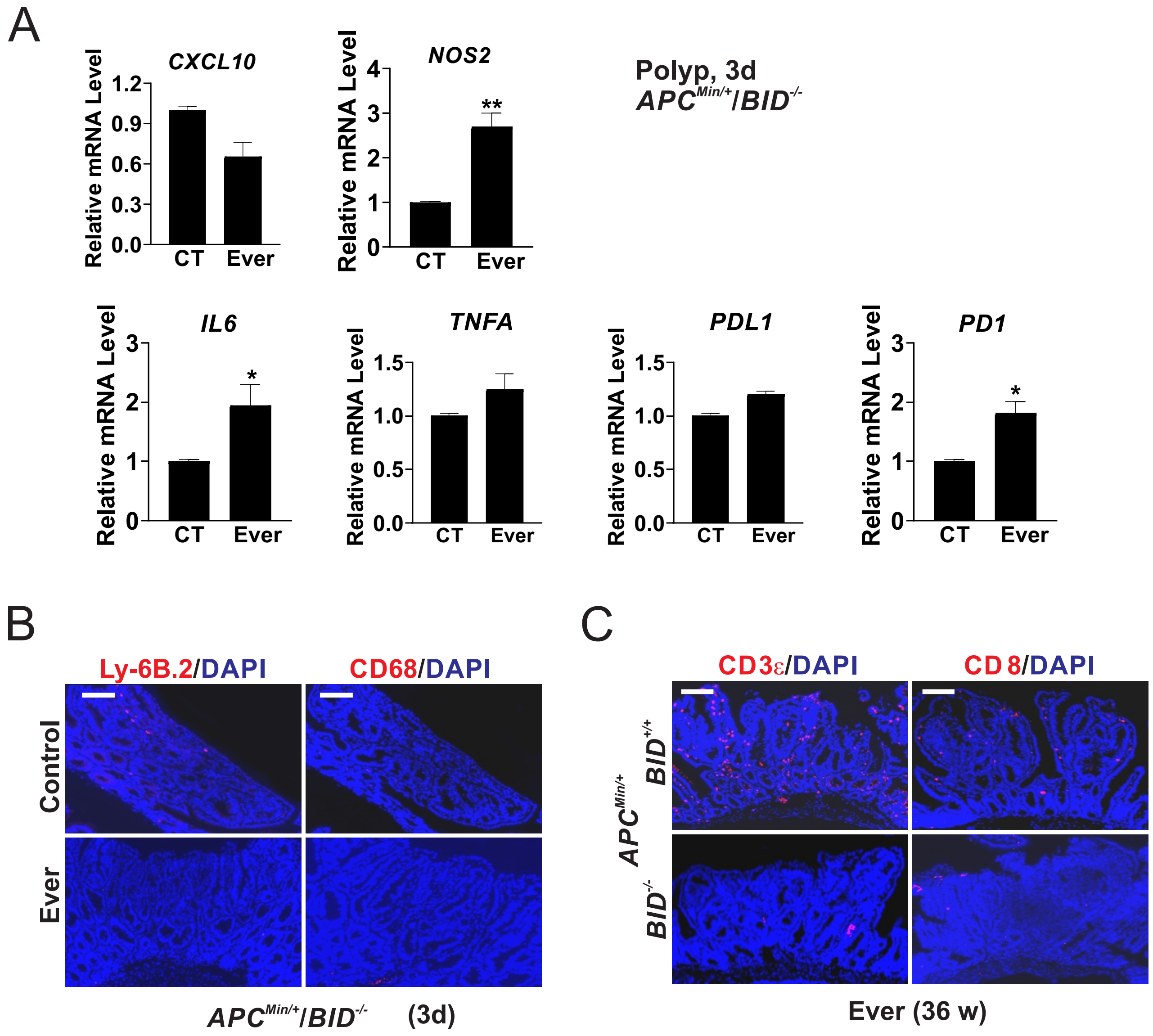
A
CXCL10
NOS2
Polyp, 3d
APCMin/+/BID-/-
Relative mRNA Level
1.2
0.9
0.6
0.3
0.0
CT
Ever
4
3
2
1
0
**
IL6
TNFA
PDL1
PD1
*
1.5
1.0
0.5
B
C
Ly-6B.2/DAPI
CD68/DAPI
Control
Ever
APCMin/+/BID-/- (3d)
CD3ε/DAPI
CD 8/DAPI
APCMin/+
BID+/+
BID-/-
Ever (36 w)
